# Increase in Pilus Islet 2–encoded Pili among *Streptococcus pneumoniae* Isolates, Atlanta, Georgia, USA

**DOI:** 10.3201/eid1606.091820

**Published:** 2010-06

**Authors:** Dorothea Zähner, Aditya Gudlavalleti, David S. Stephens

**Affiliations:** Emory University School of Medicine, Atlanta, Georgia, USA (D. Zähner, A. Gudlavalleti, D.S. Stephens); Department of Veterans Affairs Medical Center, Atlanta (D. Zähner, D.S. Stephens)

**Keywords:** Streptococcus pneumoniae, pili, streptococci, serotypes, bacteria, vaccine, research

## Abstract

To define the prevalence of pilus islet 2 (PI-2)–encoded pili in *Streptococcus pneumoniae* in a geographically defined area, we examined 590 *S. pneumoniae* isolates from population-based surveillance of invasive pneumococcal disease in Atlanta, Georgia, USA, 1994–2006. In 2006, PI-2 was present in 21% of all invasive isolates, including serotypes 1 (100%), 7F (89%), 11A (21%), 19A (40%), and 19F (75%). Only serotype 19F is included in the 7-valent pneumococcal conjugate vaccine that is in use worldwide. In 1999, PI-2-containing isolates were of the same serotypes but accounted for only 3.6% of all invasive isolates. The increase of PI-2 in 2006 resulted predominantly from the emergence of serotype 19A isolates of sequence type 320 and the expansion of serotype 7F isolates. The increase in PI-2-containing isolates and the finding that isolates of all identified serotypes expressed highly conserved PI-2 pili supports their potential as a vaccine candidate.

## Abstract 2

PI-2–encoding isolates are potential vaccine candidates.

*Streptococcus pneumoniae* is a major human pathogen, which causes pneumonia, sinusitis, otitis, bacterial meningitis, and septicemia. Introduction of the 7-valent pneumococcal conjugate vaccine (PCV7, Prevnar; Wyeth, Madison, NJ, USA) in 2000 has dramatically decreased invasive pneumococcal disease in the United States in children and in adults (*1*) and is now in use worldwide. PCV7 includes capsule polysaccharide serotypes 4, 6B, 9V, 14, 18C, 19F, and 23F, serotypes that were responsible for >80% of cases of invasive pneumococcal disease in the United States before the introduction of the conjugate vaccine (*1*). In recent years other non-PCV7 serotypes, including 7F and 19A, have been emerging in the United States, (*2*–*4*).

Pili have recently been identified on several gram-positive bacteria (*5*–*7*). To date, 2 different pilus islets (PIs) have been described in *S. pneumoniae* that encode the structural and biosynthetic genes for 2 antigenically different types of pili, PI-1 (*8*) and recently described PI-2 (*9*). The so-called *rlrA* pathogenicity islet (*10*) or PI-1 is a 14-kb genetic region present in 21%–27% of clinical isolates, depending on the geographic region analyzed. It is most prevalent in, but not restricted to, the PCV7-vaccine serotypes 4, 6B, 9V, 14, and 19F (*11*–*13*).

PI-2 is a 7-kb region located between the genes that encode peptidase T (PepT) and ferrochelatase (HemH) (*9*). The region is composed of 5 genes, which encode 2 surface proteins, PitA and PitB, with *pitA* a pseudogene due to a stop-codon in the N terminus, a signal peptidase-like protein (SipA), and 2 sortases (SrtG1 and SrtG2); the latter is nonfunctional in most of the strains (9). This PI has been reported to be present in ≈16% of the analyzed isolates belonging to serotypes 1, 2, 7F, 19A, and 19F (*9*). The presence of PI-2 pili appears to be a clonal property, rather than serotype associated, an observation that has been described for PI-1 as well (*11*–*13*). For isolates of serotype 19A, PI-2–containing isolates have been shown to be associated with sequence type (ST) ST320, a clone related to the worldwide distributed multidrug-resistant serotype 19F clone Taiwan^19F^-14 (*4*,*14*). ST320 belongs to clonal complex (CC) CC271 (with ST271 as the predicted founder of this CC); isolates of this CC have been shown to encode both PIs and express both pili concomitantly on their surface (*9*).

The PI-2 pilus is composed of polymers of the major pilus protein PitB (*9*). In contrast to other pili in gram-positive bacteria that contain accessory pilus proteins, that serve as adhesins (*15*–*17*) or adaptors for pilus attachment to the cell wall (*18*,*19*), PI-2 pili appear to consist solely of PitB polymers, and these polymers themselves have been shown to mediate adhesion of *S. pneumoniae* to eukaryotic cells (*9*).

Bagnoli et al. identified PI-2–containing isolates in a random, worldwide collection of pneumococcal isolates (*9*). We describe the distribution of PI-2 pili in a well-documented, comprehensive collection from a defined geographic region, i.e., in invasive pneumococcal isolates collected as part of a population-based surveillance program conducted in the metropolitan area of Atlanta, Georgia, USA.

## Materials and Methods

### Strain Collection

A set of 590 strains of *S. pneumoniae* from population-based surveillance of invasive pneumococcal disease isolated between 1994 and 2006 in the 8-county metropolitan Atlanta area, Georgia Health District 3 formed the basis for this study. The isolates were obtained as a part of the Centers for Disease Control and Prevention–sponsored Active Bacterial Core surveillance of the Georgia Emerging Infections Program (*1*,*20*). Information about serotype and antimicrobial drug susceptibility was determined for all isolates as described (*21*). Only viable strains with documented serotype and available antibiogram were included in the study. Laboratory strain *S. pneumoniae* R6 is a nonencapsulated derivative of the serotype 2 strain D39 (*22*).

### PCR-based Screening for PI-2

To ascertain whether PI-2 was absent from the described insertion site (*9*), we designed primers pepT_F and hemH_R (Table 1) against the flanking genes *pepT* and *hemH*. To determine whether PI-2 was present, we used primers sipA_up and sipA_dn (Table 1) designed against the PI-2–specific gene *sipA*. We tested for PI-1 using primers Rlr_up_F and Rlr_do_R for its absence and Rlr_SrtC_F and Rlr_srtD_R for its presence, respectively (Table 1).

**Table 1:** Primers used in this study of invasive Streptococcus pneumoniae isolates, Atlanta, Georgia, USA, 1994–2006*†

Region	Primer	Sequence (5′ → 3′)‡
Erm cassette	erm_F	gctctagaCGTTAGATTAATTCCTACCAGTGAC
	erm_R	gctctagaCTCCATTCCCTTTAGTAACGTGTAAC
PI-2	pepT_F	TAAGAAGCGGTCCAAGAGATTTGG
	hemH_R	AATAATGGGGCTCCAAAATCAAGC
	sipA_up_F	CTCTAGGAGGGATCTTCTTTATCATC
	sipA_do_R	CTACAGCCGTTGTTCGATTGTCC
	PilA_del_3	gcaattgcccgggcctagCCTGTATAGGGATGGTTCCAAAAG
	PilA_del_2	ctaggcccgggcaattgcGCTGGGGGCAGATGATG
	Rlr_up_F	CTTCCACGAAGTTCTTTCAATGG
PI-1	Rlr_do_R	GTCTTAGAATATCATGGTTTACGTGC
	Rlr_srtC_F	GGGGAAGATTATGCGACCTT
	Rlr_srtD_R	GCTTGGCTCTGCACGGTGCC

*PI, pilus islet. †Not included are primers used for sequencing and multilocus sequence typing. ‡Lowercase letters represent bases added for mutagenesis; uppercase letters represent bases complementary to *S. pneumoniae*.

### Multilocus Sequence Typing and Clonal Complexes

Multilocus sequence typing (MLST) was performed as described (*23*). CCs were assigned by using the eBurst program (*24*) to partition the MLST database (http://spneumoniae.mlst.net). The predicted founder of a CC was assigned by eBurst to the ST with the highest number of single-locus variants.

### Construction of a *pitB-*Deletion Mutant

The 3′- and 5′-flanking regions of *pitB* were amplified by using primer pairs sipA_up_F/PilA_del_3 and PilA_del_2/SrtC_do_R (Table 1). Both PCR products were used in a PCR-based overlap-extension reaction, which introduced an *Mfe*I restriction site between the 2 fused fragments. The PCR product was cloned into pCR-Blunt II (Invitrogen, Carlsbad, CA, USA), resulting in pCR-*pitB*. The erm-cassette of pSK-erm (*25*) was amplified by using primers erm_F and erm_R (Table 1) and ligated into *Mfe*I-digested pCR-*pitB*. The resulting plasmid, pCR-*pitB*:erm, was used to transform GA41070 according to established protocols (*26*), which resulted in mutant GA41070Δ*pitB.*

### Production of Recombinant PitB and Antiserum

The region of *pitB* that encoded the mature protein (bp 126–bp 1128) was amplified from chromosomal DNA of serotype 1 strain GA19686 and cloned into the *Escherichia coli* expression vector pET-32a+ (Stratagene, La Jolla, CA, USA). The thioredoxin/6xHis-PitB fusion protein was purified by using the B-PER 6xHis Fusion Purification Kit (Pierce, Rockford, IL, USA). The N-terminal thioredoxin/6xhis-tag was removed by cleavage with recombinant enterokinase (Novagen, Madison, WI, USA) according to the manufacturer’s recommendations. Purified PitB was used to produce a rabbit polyclonal antiserum (Covance Research Products Inc., Princeton, NJ, USA).

### Preparation of Cell Wall Extracts from *S. pneumoniae*

Cultures were grown to mid-exponential phase (optical density_600_ 0.5–0.6) in Todd-Hewitt medium containing 0.5% yeast extract. After centrifugation, the cell pellets were resuspended in 1/20 of the culture volume in extraction buffer (50 mmol/LTris-HCl; pH 8.0) containing 200 U/mL mutanolysin (Sigma-Aldrich, St. Louis, MO, USA) and 30% raffinose. After 2 h of incubation at 30°C, cell wall extracts were collected by centrifugation at 12,000 × *g* for 15 min; 20 μL cell wall extract were mixed with sodium dodecyl sulfate–polyacryamide gel electrophoresis sample buffer and boiled for 10 min immediately before separation on a 4%–12% Tris-HCl polyacrylamide gel (NuPAGE; Invitrogen). For immunostaining, proteins were transferred to a nitrocellulose membrane and detected with polyclonal anti-PitB antiserum at a dilution of 1:20,000.

### Statistical Analysis

Proportions were compared by using the Fisher exact test. p values <0.005 were considered significant.

## Results

### PI-2 in Strains of Serotypes 1, 7F, 11A, 19A, and 19F

To determine the post PCV-7 prevalence of PI-2 in *S. pneumoniae,* we analyzed all 381 viable and documented invasive isolates collected in 2006 in metropolitan Atlanta (381/409) in a PCR-based screen. Two PCRs were performed with each strain; 1 resulted in a PCR product in strains that lacked PI-2, and a second resulted in a product only in strains that contained PI-2. Strains that produced a PCR product for PI-2 were further characterized by amplification of *pitB*, which encoded the pilus backbone protein. For all strains, 1 of the 2 PCRs resulted in a PCR product. No strain gave a PCR product for both PCRs, which could have indicated the presence of the PI in an alternative integration site. These results strongly suggest that the *pepT-hemH* intergenic region is the only integration site of PI-2 in *S. pneumoniae*. Overall, 21% of all viable isolates collected in 2006 contained PI-2. The PI-2 of *S. pneumoniae* strain GA47784 was assigned the GenBank accession no. GU256423.

**Figure 1:**
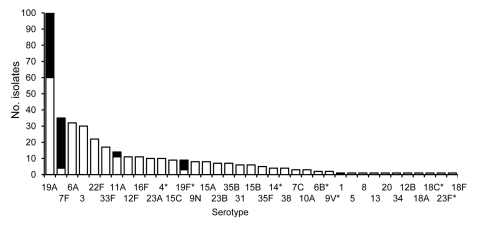
Frequency of pilus islet 2 (PI-2)–containing *Streptococcus pneumoniae* invasive isolates in metropolitan Atlanta, Georgia, USA, 2006. Black, PI-2–containing isolates; white, PI-2–lacking isolates. *Serotypes included in the 7-valent pneumococcal conjugate vaccine.

PI-2 was identified only in isolates of serotypes 1, 7F, 11A, 19A, and 19F (Figure 1). Serotypes 19A and 7F were the most frequent PI-2–containing serotypes isolated in 2006, with a PI-2 prevalence of 40% and 89%, respectively. In serotype 11A, PI-2 was identified sporadically, whereas for serotype 19F, 75% of the isolates contained PI-2. Serotype 1 was represented by a single isolate in 2006. To determine the prevalence of PI-2 in serotype 1 isolates, we analyzed an additional 20 invasive isolates, collected in Atlanta from 1994 through 2005; all contained PI-2. Serotype 1 had the highest presence of PI-2 of all analyzed serotypes. Except for serotype 19F, the PI-2–containing serotypes are not included in PCV7.

To further characterize PI-2 of a serotype 11A strain, we sequenced the 7-kb region between the flanking genes *pepT* and *hemH* of strain GA47784. The sequence was 99.9% identical to the published PI-2 sequences, with the highest sequence identity to serotype 1 strain PN110, with only 1-bp difference over the entire PI-2 region. The mutation was located downstream of the stop-codon in *pitA,* which results in premature termination of PitA, as has been reported (*9*).

### Effects of PCV7 Introduction on PI-2 Distribution

**Figure 2:**
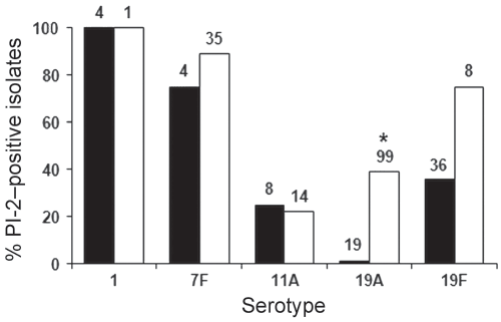
Percentage of pilus islet 2 (PI-2)–containing *Streptococcus pneumoniae* invasive isolates among serotypes associated with PI-2 in metropolitan Atlanta, Georgia, USA, 1999 and 2006. The total number of isolates for each serotype is shown at the top of the column. *Significant difference between 1999 and 2006 19A isolates (p<0.005).

To assess whether PI-2 was associated with the emergence of serotypes in the aftermath of PCV7 introduction (2000), the PI-2 distribution was determined in invasive isolates from 1999 that belonged to the PI-2–containing serotypes (Figure 2). The numbers of isolates for the different serotypes in the 2 periods reflected the expansion of isolates of the nonvaccine serotypes 7F and 19A and the decline in 19F isolates (Figure 2) that has been described in the United States since the introduction of PCV7 (*4*). PI-2 was present in isolates of all 5 serotypes before the introduction of PCV7; hence, PI-2 was not recently acquired. For serotype 7F, the ratio between PI-2–containing and PI-2–lacking isolates in 2006 compared to the ratio in 1999 remained essentially the same (Figure 2), which suggests that PI-2 pili may not provide a major selective advantage to invasive 7F isolates. Serotype 19A showed a significant increase in PI-2–containing isolates. To assess the presence of PI-2 in other serotypes in 1999, we analyzed isolates of all represented serotypes of PI-2: all isolates of infrequent serotypes (1, 5, 7C, 7F, 8, 9A, 9N, 10A, 13, 15B, 15C, 16, 16F, 18F, 20, 23A, 24B, 31, 33F, 34, 35B, 35F, 37) and at least 10% of the isolates of serotypes with >10 isolates (3, 4, 6A, 6B, 9V, 11A, 12F, 14, 18C, 19A, 19F, 22F, 23F). No additional PI-2–containing isolates were detected, which suggests that serotypes 1, 7F, 11A, 19A, and 19F were the only serotypes that contained substantial numbers of PI-2–containing isolates. These PI-2–containing isolates accounted for ≈3.6% of all viable isolates collected in 1999. The estimation of total viable isolates in 1999 was based on the known proportion of viable isolates to total collected isolates of the PI-2–containing serotypes of 1999 (93%).

### Genetic Relationships Between PI-2–containing Strains

To analyze the clonality of the PI-2–containing isolates, we performed MLST for all PI-2–containing isolates from 1999 and 2006 (Table 2). For serotype 1, the isolates from 1999 and 2006 belonged to ST304 and ST227, respectively (Table 2). Both STs have been described as the most prevalent serotype 1 STs in the United States (*27**,**28*). PI-2–containing serotype 7F isolates of 2006 belonged to either ST191 or ST1176, and ST191 isolates were responsible for the strong expansion of this serotype in 2006. 7F isolates lacking PI-2 were tested and found to be of ST191 (data not shown), which indicated that PI-2 is present in only a subpopulation of this ST. ST1176 is unrelated to ST191, and 7F isolates of this ST were not present in 1999. All PI-2–containing isolates of serotype 11A belonged to ST62. Analysis of additional serotype 11A isolates that lacked PI-2 showed that they all belonged to ST62 (data not shown). This finding suggests that the structure of this serotype is clonally homogeneous and that PI-2 is present in a subset of this ST.

**Table 2:** Clonal distribution of PI-2-containing *Streptococcus pneumoniae* isolates, Atlanta, Georgia, USA, 1994–2006*

Serotype	CC	ST	No. PI-2-containing isolates	
			1999	2006
1	304	304	1	1
	306	227 (DLV)	3	–
7F	191	191	3	28
	218	1176 (DLV)	–	3
11A	53	62	2	3
19A	2090	1339 (SLV)	1	9
		2268 (DLV)	–	3
	271	320 (SLV)	–	27
19F	271	271	1	–
		236 (SLV)	3	1
		3039 (SLV)	–	1
		651 (DLV)	3	2
	251	251	1	1
		654 (SLV)	4	1
		1258 (DLV)	2	–

*CC, clonal complex; ST, sequence type; PI, pilus islet; DLV, double-locus variant; SLV, single-locus variant.

For serotype 19A, 27 of the 39 PI-2–containing 19A isolates in 2006 were ST320 (Table 2). This ST was absent in 1999 (*29*); thus these isolates were the major contributor to the increase of PI-2–containing isolates of serotype 19A in 2006. Additionally, PI-2 was identified in 19A isolates of ST1339 and ST2268, and both sequence types belonged to CC2090. One ST1339 isolate was present in 1999, which indicated that PI-2 was present in this ST before PCV7 was introduced. 19A isolates of 2006 that lacked PI-2 were tested and found to belong to other STs, e.g., ST199 and ST695, STs consistent with the described population structure of 19A isolates in the United States (*4*).

ST analysis of the remaining isolates of 19F isolates in 2006 showed that PI-2 was present in isolates that belonged to CC271 (ST236, ST651, ST3039) and also in the unrelated CC251 (ST251, ST654, ST1258) (Table 2). However, analysis of several 19F isolates that lacked PI-2 revealed isolates of ST3039 (data not shown); this finding indicated either a loss of PI-2 or a recent acquisition of the islet in a subset of isolates of this ST. Isolates of CC271 have been described to encode and express PI-1 and PI-2 concomitantly (*9*). Analysis for the presence of PI-1 in our population confirmed PI-1 in isolates of CC271 (ST320, ST271, ST3039, ST236, ST651). Additionally, PCR results showed that PI-1 was in ST1339 isolates but not in isolates of the single locus variant ST2268. Isolates of all other PI-2–containing serotypes or STs did not contain PI-1.

### Antimicrobial Drug Resistance and PI-2–containing Isolates

19F and 19A isolates of CC271 are closely related to the globally distributed multidrug-resistant clone Taiwan^19F^-14, and all were resistant to penicillin, erythromycin, cotrimoxazole, tetracycline, and chloramphenicol. In addition, serotype 19A isolates of CC2090 (ST1339, ST2268) were resistant to penicillin, erythromycin, and cotrimoxazole. All PI-2–containing 19A isolates in 2006 were multidrug resistant. In contrast, PI-2–containing isolates of serotypes 1, 7F, 11A, and 19F (ST654, ST251) were susceptible to all tested antimicrobial drugs. Hence, antimicrobial drug resistance was not a consistent feature of PI-2–containing isolates.

### Expression of High Molecular Weight PitB on the Surface of PI-2–containing Isolates

**Figure 3:**
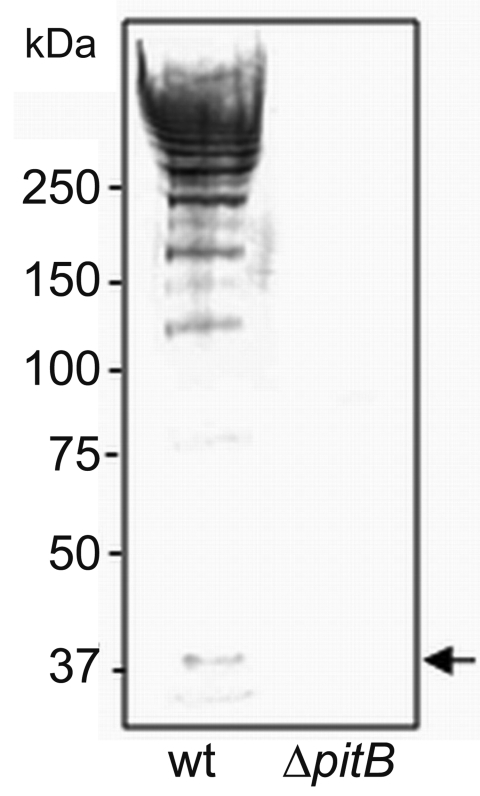
Detection of high molecular weight PitB polymers in invasive isolates of *Streptococcus pneumoniae*. Western blot of cell wall extracts from strains GA41070 (lane 1) and GA41070Δ*pitB* (lane 2) detected with anti-PitB antiserum. Monomeric PitB (arrow) and the marker sizes are indicated.

The first report of PI-2 pili described mutations in several genes encoded by PI-2 (namely, *pitA* and *srtG2*), which resulted in their inactivation (*9*). To confirm that PI-2–containing isolates indeed express PitB polymers on their surface, we produced an anti-PitB antiserum to detect PitB polymers. The specificity of the PitB antiserum was tested against cell wall extracts of strain GA41070 and its isogenic *pitB* deletion mutant, GA41070Δ*pitB* (Figure 3). In the wild type, the antibody reacted with several bands that produced the high molecular weight (HMW) banding pattern characteristic of gram-positive pili (*30*,*31*) (Figure 3). As expected, the PitB monomer and the HMW banding pattern were absent in the *pitB-*deletion mutant (Figure 3).

**Figure 4:**
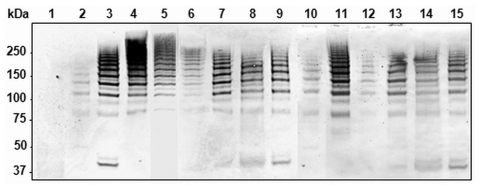
Detection of high molecular weight PitB polymers in invasive isolates of *Streptococcus pneumoniae*. Western blot of cell wall extract from strains R6 (lane 1), GA47901 (lane 2), GA13444 (lane 3), GA47077 (lane 4), GA47340 (lane 5), GA47784 (lane 6), GA47368 (lane 7). GA47751 (lane 8), GA47187 (lane 9), GA11293 (lane 10), GA47628 (lane 11), GA49138 (lane 12), GA47434 (lane 13), GA47373 (lane 14), and GA47105 (lane 15) detected with anti-PitB antiserum. Marker sizes are indicated.

To analyze whether the PI-2–containing isolates of the different STs produced PitB polymers, we used Western blots to examine their cell wall extracts (Figure 4). All PI-2–containing strains showed the same HMW banding pattern (Figure 4, lanes 2–15). In addition, all 81 PI-2–containing isolates from 2006 were analyzed by whole cell dot blots that confirmed that all isolates expressed PitB on their surface (data not shown).

### High Conservation of PitB among Different STs

To determine the degree of variation in PitB, we sequenced *pitB* in isolates representing the different STs (Table 2). The *pitB* sequences were 100% identical for all strains, except the 7F isolates GA47077 (ST191) and GA47340 (ST1176), which have a single point mutation that has been observed in other serotype 7F strains (*9*). The high conservation of PitB was consistent with the reported PitB sequences by Bagnoli et al. (*9*) and is an essential feature of PI-2 as a potential vaccine candidate. Additionally, we analyzed the gene for the second surface protein encoded in PI-2, *pitA,* and confirmed the presence of the described stop-codon in all strains representing the different STs (Table 2).

## Discussion

PI-1 pili on *S. pneumoniae* have been reported as promising vaccine candidates for this important human pathogen (*13*,*32*). One limitation to this approach is that PI-1 has been found in only ≈25% of strains; it is most frequently found in pneumococcal isolates of serotypes covered by PCV7, which are decreasing in countries that have introduced the vaccine. The recent identification of PI-2 in serotypes not covered by PCV7 has raised the possibility that broad coverage of strains can be achieved by a bivalent pilus-based vaccine.

The first report on PI-2 pili described the serotypes and sequence types associated with PI-2 (*9*). In the current study, we defined the prevalence of PI-2–expressing isolates in a defined geographic region and population and assessed whether the introduction of PCV7 had an effect on the distribution of PI-2–containing isolates. Twenty-one percent of the 381 viable invasive pneumococcal isolates collected in 2006 in metropolitan Atlanta expressed PI-2 pili. This percentage is higher than the PI-2 incidence reported from a global strain collection (*9*) and is similar to the reported percentage for PI-1 (*11*–*13*). The higher incidence rate of PI-2 in our study refers to a post-PCV7-era population, whereas the earlier report was based on a random strain collection, including pre- and post-PCV7 isolates. Therefore, our higher results are consistent with the assessment of Bagnoli et al. (*9*) that PI-2 is most prevalent in emerging, non-PCV7 serotypes.

Notably, all serotype 1 strains had PI-2. This serotype does not play a large role in pneumococcal invasive disease in the United States (*27*), a conclusion reflected by the low number of serotype 1 isolates in our dataset. However, serotype 1 isolates have been identified in several studies as particularly invasive, in contrast to other serotypes that are more frequently associated with carriage (*33*–*35*) and associated with epidemic outbreaks (*27*,*36*,*37*). Further study of PI-2 prevalence in serotype 1 requires a geographically different dataset with a higher serotype 1 prevalence.

In contrast to serotype 1, serotype 7F, together with 19A, has been a major emerging serotype in the post-PCV7 era (*3*,*4*), a fact reflected in our dataset, with a 5-fold increase between 1999 and 2006. Serotype 7F causes primary invasive disease in otherwise healthy patients and is found less frequently in carriers (*33*,*35*,*38*). As most of the serotype 7F isolates belong to ST191, the genetic structure of this serotype is very homogeneous (*35*). However, 3 isolates of ST1176 were found in 2006. ST1176 belongs to CC218 and is unrelated to ST191. A 7F isolate of CC218 had been observed in 1999 (*29*), a CC previously associated with isolates of serotype 12F. The ratio between PI-2–containing and –lacking isolates of serotype 7F was essentially unchanged from 1999 to 2006; only the overall number of cases increased. Thus, PI-2 does not appear to provide a selective advantage to invasive serotype 7F isolates, i.e., contribute to the emergence of this serotype.

Serotype 11A has been described as an opportunistic serotype often found in asymptomatic carriers and a cause of disease in patients with underlying disease (*33*,*35*). However, when serotype 11A does cause invasive disease, it has been associated with a high number of deaths (*35*). Isolates that cause invasive disease have been identified as belonging to ST62, the ST most common among serotype 11A (*35*). The low prevalence of PI-2 in serotype 11A suggests that PI-2 pili are not essential for isolates of this serotype to cause invasive disease.

The genetic profile of the serotype 19A isolates in Atlanta is consistent with the described clonal distribution of this serotype in the United States in the post-PCV7 period (*4*). Several capsular switching events have contributed to the emergence of serotype 19A, including (but not limited to) ST320, originating from a multidrug-resistant derivative of clone Taiwan^19F^-14(ST236), and CC2090 (ST1339, ST2289) from the highly related clone North Carolina^6A^-23 (*39*). Unlike serotype 7F, 19A is characterized by a heterogeneous clonal structure (*4*), and the presence of PI-2 is a feature of some of the clones.

Most of the PI-2–containing isolates that we found do belong to the serotypes reported for PI-2 by Bagnoli et al. (*9*). Serotype 2 was absent from our population during the surveillance period of 1994 to 2006, and in addition we identified PI-2 in serotype 11A. Overall, we confirmed the presence of PI-2 in emerging non-PCV7 serotypes. However, other non-PCV7 serotypes that do not have PI-2 are emerging as well, e.g., 15BCF, 22F, 33F, and 38 (*2*,*40*), which indicates that other mechanisms may influence the emergence of new serotypes.

Of note, 3 of the 4 non-PCV7 serotypes with PI-2–containing isolates (serotypes 1, 7F, 19A) are included in the 13-valent pneumococcal conjugate vaccine now being introduced in Europe, Canada, and the United States. Use of this vaccine is expected to greatly reduce the frequency of all included serotypes and may thus reduce the proportion of *S. pneumoniae* isolates that contain PI-2. In summary, the prevalence of PI-2 increased from ≈3.6% of the invasive pneumococcal isolates in 1999 to 21% in 2006 in metropolitan Atlanta, Georgia, especially in the emerging serotypes 7F and 19A. PI-2-containing isolates of all identified sequence types expressed polymers of the highly conserved pilus protein PitB on their surface. These findings support the potential of PI-2 pili as a vaccine candidate.
